# Levels of well-being according to demographic variables in Saudi Arabia: a PERMA model survey study

**DOI:** 10.3389/fpsyg.2025.1538986

**Published:** 2025-04-01

**Authors:** Monira A. Almeqren, Aljawharh Alsukah, Aljawharah Almuqrin, Ebtesam A. Alzeiby, Ali F. Alamri, Munirah Alshebali, Nourah A. AlGadheeb

**Affiliations:** ^1^Department of Psychology, College of Education and Human Development, Princess Nourah bint Abdulrahman University, Riyadh, Saudi Arabia; ^2^Department of Health Sciences, College of Health and Rehabilitation Sciences, Princess Nourah bint Abdulrahman University, Riyadh, Saudi Arabia; ^3^Department of Family Medicine, King Abdullah bin Abdulaziz University Hospital, Riyadh, Saudi Arabia; ^4^Department of Technical Sciences Programs, Applied College, Princess Nourah bint Abdulrahman University, Riyadh, Saudi Arabia

**Keywords:** well-being, PERMA model, demographic variables, Saudi Arabia, survey study

## Abstract

**Objective:**

This study aims to assess the level of well-being among the Saudi population based on their demographic variables.

**Methods:**

The sample comprised 2,927 individuals from 13 regions of the Kingdom, selected through a stratified random sampling method. The study utilized the PERMA model of well-being as its primary tool. The findings revealed that the participants’ average scores on the well-being scale ranged from 5.035 to 7.879, with an overall arithmetic mean of 6.569, indicating a high level of well-being.

**Results:**

The results showed no significant gender-based disparities in well-being levels. However, variations were observed across different educational levels. The study also found consistently high levels of well-being across all sub-categories of nationality, income, and family size.

**Conclusion:**

The researcher recommends that officials in Saudi Arabia continue their efforts to enhance the psychological well-being of individuals by implementing diverse activities and programs aimed at promoting psychological safety and improving overall well-being.

## 1 Introduction

The field of positive psychology has expanded significantly over the past two decades, and for good reason. Research indicates that subjective well-being and related concepts, such as life satisfaction, happiness, and optimism, have numerous positive effects on health, education, success, and other critical life outcomes (Diener et al., 2018; Rüppel et al., 2015; Tay et al., 2014). The term “well-being” broadly encompasses all the ways individuals experience and evaluate their lives positively. In essence, this means that individuals can experience life positively in diverse ways (Park et al., 2023). While some equate well-being with happiness, this association can evoke images of cheerfulness and joyfulness that may not resonate with everyone. Consequently, some prefer to conceptualize well-being as a prolonged state of contentment. For others, well-being is primarily about having good physical and mental health (Ruggeri et al., 2020; Tov, 2018). The World Health Organization (WHO) defines well-being as “a mental state characterized by the ability to realize one’s capabilities, engage in productive and creative work, and effectively manage the typical challenges and pressures of life” (World Health Organization, 2022). It is understood as the outcome of a harmonious psychological process that provides individuals with a sense of meaning and purpose in life, rather than merely the absence of negative emotions (Dhanabhakyam and Sarath, 2023; Dr and Shukla, 2023).

According to Seligman’s theory of well-being, well-being is an abstract concept that includes feeling satisfied and doing well, a high level of mental health, and is also presented as a guide to helping individuals thrive (Seligman, 2011). Seligman, on his side, describes well-being using the five core dimensions of PERMA, which include the concepts of positive emotion (P), engagement (E), relatedness (R), meaning (M), and accomplishment (A) (Kovich et al., 2023). That is, positive emotion includes hedonic feelings, such as happiness, pleasure, and well-being, Engagement represents the effort to participate and indulge in activities that are intrinsically satisfying, the loss of feelings of self-consciousness, and the experience of a sense of time standing still. To explain further, relatedness emphasizes high-quality relationships with others and integration with society or community, accomplishment is seen as representing accomplishment, success, or mastery of tasks, Meaning is defined as belonging to something larger than oneself and a sense of connection to something beyond oneself. While the multidimensional construct is thought to provide a more in-depth overview of well-being and more specific targets (i.e., dimensions) for potential improvements (Feng et al., 2020; Hone et al., 2014; Umucu et al., 2021; Wagner et al., 2020).

The factors influencing welfare outcomes in Arab countries differ from those in Western or Asian literature due to cultural, social, political and religious factors unique to the region (Sweileh, 2021). Arab societies often emphasize strong family ties, communities, and collective values, which can influence perceptions and experiences of well-being (Al-Hendawi et al., 2024). In addition, diverse socio-political landscapes and economic conditions in Arab countries can influence mental health and social attitudes toward well-being. Moreover, religion plays a central role in Arab countries, affecting daily life and mental health prospects (Al-Hendawi et al., 2024; Sweileh, 2021).

The present study’s context is Saudi Arabia, which has undergone social, economic and cultural transformation in the past two decades (AlzaidAlsharif, 2022). These developments are bound to affect members of society, especially students, who differ significantly from previous generations in many ways, including improving living standards and increasing options to meet their needs. In addition, there is considerable pressure to deal with peer pressure, social norms and high standards of success (AlzaidAlsharif, 2022; Nurunnabi, 2017). in fact, success is closely linked to life satisfaction and positive emotions; therefore, understanding students’ perspectives and emotional states is essential to build interventions that may improve their well-being (Gill et al., 2021). To develop effective positive psychology programs and interventions by individuals, especially students, stakeholders, specialists and teachers must first understand the factors that drive the desired positive outcomes for students and other members of society in general (Al-Hendawi et al., 2024; Tansey et al., 2018).

Add to all what have been afore-said, demographic factors such as gender, age, education, employment, marital status, financial income, family size, and nationality are suggested to influence well-being in various ways (Fan and Babiarz, 2019; Melero et al., 2023; Owusu, 2023; Thomson et al., 2018). For example, age and financial satisfaction are found to enhance psychological well-being (Owusu, 2023). That is, older individuals who are earning more money tend to experience higher levels of psychological well-being than their younger counterparts (Owusu, 2023). Also, the results showed few gender differences in psychological well-being, although women reported experiencing positive and negative emotions more frequently and intensely than men (Matud et al., 2019). Although the literature has shown differences between women and men in some dimensions of psychological well-being (Ahrens and Ryff, 2006; Karasawa et al., 2011; Lindfors et al., 2006), these differences generally vary depending on other factors such as age, culture, or the roles that an individual plays (Karasawa et al., 2011). Moreover, higher levels of education are also argued to correlate with higher levels of psychological well-being (Yafi et al., 2021).

While understanding the importance of conducting studies assessing awareness of emotional well-being, specific age groups, people with chronic diseases, and mental health during disease epidemics (Abu-Helalah et al., 2022; Al Mutair et al., 2021; Hamdan-Mansour et al., 2015), this limited amount and scope of research does not allow for an understanding of mental health levels and challenges, the impact of demographic variables, and comparisons with other countries. The primary aim of this study was to measure the level of psychological well-being in Saudi Arabia. The secondary aim was to measure the influence of demographic variables, namely gender, age, education, employment, marital status, financial income, family size, and nationality, on well-being levels in Saudi Arabia. To achieve these objectives, the following research questions were explored:

1. What is the level of psychological well-being in Saudi Arabia?
2. Does the level of psychological well-being vary based on other variables such as genders, levels of education, nationality, income, and family size?

## 2 Methodology

### 2.1 Participant recruitment

This cross-sectional descriptive study was conducted to measure the well-being of community members in Saudi Arabia in 2024. The sample was selected using a stratified random sampling method. The stratified random selection was carried out by identifying the 13 administrative regions that make up the Kingdom of Saudi Arabia, and within each region the available method and the snowball method were used to reach the different samples, taking into account reaching samples from different categories according to the study variables. The initial sample consisted of 3,323 individuals; however, 396 participants were excluded due to incomplete responses, resulting in a final sample size of 2,927 individuals. The requisite sample for drawing a representative sample from the Saudi society was calculated using the following equation (Yamane, 1967):


$$n=N/\left(1+Ne2\right)$$

where:

- N is the number of individuals in the study community and is equal to (32,175,224) according to the General Authority for Statistics in the Kingdom.
- e represents the degree of confidence or significance level, which is: (0.05) or (0.04) or (0.03) or (0.02) or (0.01).

Using a significance level of 0.02, we used 2,500 as the target minimum sample size.

The research team chose a sample of 3,323 individuals, which is more than the required sample size of 2,500. Following the exclusion of incomplete responses, the final sample of 2,927 was still larger the recommended 2,500 to fully represent the study community at the level of (0.02), i.e., at a confidence level of 98%, which is a high confidence level. The general rule for samples is that increasing the sample size reduces the sampling error and can provide a higher representation of the characteristics of the community and thus a more accurate generalization of the research results (Hassan, 2016). Thus, our sample was adequately representative for a nationwide sample by the above guidelines. Furthermore, when applying the sample size calculation equation approved by the American Education Association (Morgan, 1970), we would get almost the same sample size.

With reference to participant characteristics in the final sample, the mean age of participants was 24.94 years (SD = 8.41). Table 1 provides detailed demographic information about the participants.

**Table 1:** Demographic characteristics of the study participants (*n* = 2,927).

Demographic characteristics		*N*	Percentage
Gender	Male	880	30.1%
	Female	2047	69.9%
Age	15–24	1,305	44.6%
	25–34 years	683	23.3%
	35–49 years	725	24.8%
	50–65 years	191	6.5%
	≥66 years	23	0.8%
Education	Post university	185	6.3%
	University	1969	67.3%
	Secondary	658	22.5%
	Intermediate	72	2.5%
	Primary	23	0.8%
	Uneducated	20	0.7%
Income	≤15,000 Riyal Saudi	1862	63.6%
	15,001–25,000 Riyal Saudi	612	20.9%
	≥25,001 Riyal Saudi	136	4.6%
	Other	317	10.8%
Job	Working	1,107	37.8%
	Not working	562	19.2%
	Students	1,142	39%
	Retired	116	4%
Marital status	Married	1,186	40.5%
	Divorced	85	2.9%
	Widower	26	0.9%
Nationality	Saudi	2,734	93%
	Non-Saudi	193	7%
Family size	1–2	291	9.9%
	3–4	577	19.7%
	≥ 5	2059	70.3%

### 2.2 Data collection procedure

Data were collected in March 2024. Thirty-five research assistants were recruited from 13 regions of the Kingdom, and they were trained on the method of applying the scale. They supervised the process and ensured that each individual in the sample completed the scale. It took about 15 min to complete the scale, and data collection took 6 months. Before data collection, potential participants were informed via social media about the opportunity to volunteer for the study. They were provided with a link containing detailed research information and the survey link. Participants were notified that completing the questionnaire would indicate their consent to participate voluntarily. It was explicitly stated that all information obtained would remain confidential and be used solely for research purposes. The survey included demographic information and the PERMA scale, detailed below.

The study received ethical approval from the Institutional Review Board (IRB) of King Abdulaziz City for Science and Technology (IRB registration number HAP-01-R-059; log number 22-0275) and the Scientific Research Deanship at Princess Nourah bint Abdulrahman University. All research procedures adhered to the Helsinki Declaration. Data collection was completed within 2 months.

### 2.3 Data collection tools

#### 2.3.1 The PERMA scale

The PERMA scale is a widely used measure of subjective well-being. The scale is grounded in Martin Seligman’s PERMA model, which identifies five axes of well-being: Positive Emotion, Engagement, Relationships, Meaning, and Accomplishment. The scale comprises 23 questions addressing health, negative feelings, loneliness, and general happiness, organized into seven groups reflecting the PERMA dimensions. Responses are recorded on an 11-point Likert scale, ranging from 0 (“never,” “terrible,” “not at all”) to 10 (“always,” “excellent,” “completely”).

The original scale has demonstrated strong psychometric properties in previous studies (Ayse, 2018 Goodman et al., 2018 de Carvalho et al., 2023). For this study, the PERMA scale was translated into Arabic and then back-translated into English by language experts to ensure linguistic and conceptual consistency with the original. First, a bilingual professor translated the English version into Arabic. Then, a second professor, a native Arabic speaker specializing in English, re-translated it into English. Finally, three specialists in Arabic, psychology, and English reviewed both versions. Based on their consensus, minor revisions were made to create the final Arabic version. The scale posed no difficulties for use in the Saudi context. In this study, the Arabic version of the PERMA scale demonstrated excellent reliability, with Cronbach’s alpha values ranging from 0.70 to 0.83.

### 2.4 Data analysis

**Figure 1:**
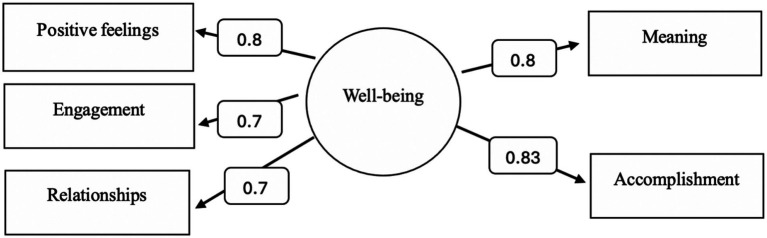
Confirmatory factor analysis model for a measure of well-being with a single latent factor.

All statistical analyses were conducted using SPSS version 20. The internal consistency of the PERMA scale was assessed by calculating the correlation of each item with the total score of its respective dimension and the overall scale score. Correlation coefficients were classified as follows: r = 0.00–0.30 (negligible), r = 0.30–0.50 (weak), r = 0.50–0.70 (moderate), and r > 0.70 (strong). Confirmatory Factor Analysis (CFA) was performed to validate the factorial structure of the PERMA scale for the study sample (*N* = 2,927). The CFA model assumed a single latent construct representing well-being, encompassing the five observed dimensions of the PERMA framework. The model exhibited strong goodness-of-fit indices, with a non-significant chi-square (χ^2^) value and an expected false validity index lower than that of the saturated model. Additional indices, including the Comparative Fit Index (CFI), Tucker-Lewis Index (TLI), and Root Mean Square Error of Approximation (RMSEA), also indicated a good fit, demonstrating the scale’s structural validity (Figure 1).

The reliability of the PERMA well-being scale for the total sample was assessed using Cronbach’s alpha for the sub-dimensions and the total scale score. Additionally, reliability was evaluated using the split-half method, with the correlation coefficient calculated between the two halves of the scale. The Guttman split-half reliability coefficient was also computed. Since the scores of the two halves of the scale are unequal, Table 2 presents the reliability values derived from both Cronbach’s alpha and the split-half method.

**Table 2:** *T*-test analysis of level of well-being based on nationality (*n* = 2,927).

Dimensions	Saudi nationality (*n* = 2,734)		Non-Saudi nationality (*n* = 193)		*T*	*Sig.*
	*M*	SD	*M*	SD		
Positive feelings	20.493	6.568	20.626	6.490	0.274	*NS*
Engagement	19.903	5.974	19.709	5.812	0.436	*NS*
Relationships	20.612	6.360	20.673	6.419	0.129	*NS*
Meaning	20.801	6.828	21.062	6.894	0.513	*NS*
Accomplishment	19.892	5.870	19.663	5.905	0.523	*NS*
Total score	151.133	31.297	150.813	32.251	0.137	*NS*

NS, not significant.

All validity coefficients, representing the loadings of the well-being aspects on the single latent factor of the scale, were statistically significant at the 0.01 level. This confirms the validity of all aspects of the well-being scale within the study sample. The reliability coefficients for the five dimensions of the scale ranged from 0.55 to 0.73, which are considered acceptable. These findings indicate the stability and consistency of the well-being scale’s dimensions in the study sample.

## 3 Results

### 3.1 Level of well-being among the population of the Kingdom of Saudi Arabia

Table 3 presents the levels of well-being among the study sample. The data were analyzed using the arithmetic mean and standard deviation. The results indicate that the overall mean score for all scale items is relatively high, with a mean of 6.569 and a standard deviation of 2.713. Among the PERMA dimensions, the highest scores were observed for meaning, relationships, and positive feelings, followed by engagement and accomplishment. Regarding the sub-dimensions, general happiness and health scored higher than negative feelings and loneliness.

**Table 3:** Arithmetic means and standard deviations for the level of well-being in the PERMA model among the population of the Kingdom of Saudi Arabia (*n* = 2,927).

Dimensions	Paragraphs	*M* (paragraphs)	SD (paragraphs)	*M* (dimension)	SD (dimension)	Dimensions	Paragraphs	*M* (paragraphs)	SD (paragraphs)	*M* (dimension)	SD (dimension)
Positive Feelings	P1	6.355	2.719	6.833	2.530	Negative Feelings	N1	5.801	2.983	5.546	2.809
	P2	6.645	2.426				N2	5.412	2.704		
	P3	7.501					N3	5.427	2.741		
Engagement	E1	6.411	2.729	6.629	2.749	Health	H1	7.478	2.378	6.854	2.671
	E2	6.739	2.707				H2	6.132	2.997		
	E3	6.739	2.813				H3	6.953	2.638		
Relationships	R1	6.154	2.995	6.872	2.939	Loneliness	Lon	5.035	3.300	5.035	3.300
	R2	7.525	2.995								
	R3	6.937	2.825								
Meaning	M1	6.982	2.573	6.939	2.630	General happiness	Hap	7.166	2.487	7.166	2.487
	M2	7.084	2.659								
	M3	6.751	2.659								
Accomplishment	A1	6.222	2.593	6.625	2.545	Overall score of well-being	6.569	2.713			
	A2										
	A3										

### 3.2 Levels of well-being according to demographic variables in the study sample

Table 4 indicates that there were no statistically significant differences between male and female groups across the five elements of well-being in the PERMA model. The calculated *t*-values ranged from 0.127 to 0.929, all of which were smaller than the critical *t*-value of 1.96 at the 0.05 significance level. These results suggest that there are no significant differences in well-being between males and females in the study sample.

**Table 4:** *T*-test analysis of level of well-being based on gender (*n* = 2,927).

Dimensions	Female (*n* = 2047)		Male (*n* = 880)		*T*	*Sig.*
	*M*	SD	*M*	SD		
Positive feelings	20.555	6.554	20.377	6.581	0.674	*NS*
Engagement	19.899	6.039	19.869	5.785	0.127	*NS*
Relationships	20.688	6.377	20.450	6.331	0.929	*NS*
Meaning	20.758	6.880	20.956	6.718	0.719	*NS*
Accomplishment	19.916	5.893	19.786	5.822	0.548	*NS*
Total score	151.129	31.259	151.070	31.595	0.927	*NS*

NS, not significant.

Table 5 reveals that there are no statistically significant differences attributable to the age variable across the five elements of well-being in the PERMA model (positive feelings, engagement, relationships, meaning, and accomplishment) or the overall well-being score. The analysis shows that the calculated *F*-values for all elements and the total score are lower than the critical *F*-values, which are 2.70 at the 0.05 significance level and 3.98 at the 0.01 significance level. These results indicate that age does not significantly influence the well-being dimensions measured in this study.

**Table 5:** One-way ANOVA analysis of level of well-being based on age (*n* = 2,927).

Variables according to age	From 15 to 24 (*n* = 1,305)		From 25 to 34 Years (*n* = 683)		From 35 to 49 (*n* = 725)		From 50–65 Years (*n* = 191)		Over 65 Years (*n* = 23)		*f*	*p*-value	*Sig.*
	*M*	SD	*M*	SD	*M*	SD	*M*	SD	*M*	SD			
Positive feelings	20.578	6.480	20.117	6.824	20.575	6.514	21.000	6.381	21.173	6.183	0.994	0.409	*NS*
Engagement	19.652	6.029	19.811	6.051	20.223	5.757	20.460	5.978	20.565	5.383	1.628	0.164	*NS*
Relationships	20.597	6.273	20.428	6.507	20.715	6.354	20.759	6.566	22.608	5.606	0.793	0.530	*NS*
Meaning	20.703	6.846	20.761	6.569	20.984	7.003	20.916	7.160	22.217	5.838	0.503	0.734	*NS*
Accomplishment	19.760	5.904	19.705	5.894	20.231	5.784	19.664	5.938	21.565	5.159	1.542	0.187	*NS*
Total score	150.264	31.326	150.831	31.650	152.282	31.424	152.403	30.415	157.043	30.282	0.835	0.503	*NS*

NS, not significant.

Table 2 demonstrates that there are no statistically significant differences in the five elements of well-being in the PERMA model (positive feelings, engagement, relationships, meaning, and accomplishment) or the overall well-being score between Saudi and non-Saudi participants. The calculated *t*-values ranged from 0.129 to 0.523, all of which are smaller than the critical *t*-value of 1.96 at the 0.05 significance level. These results indicate no significant differences between Saudi and non-Saudi participants in any of the five elements of well-being or the overall well-being score as measured by the PERMA model.

Table 6 shows that there are no statistically significant differences attributable to the academic level variable across the five elements of well-being in the PERMA model (positive feelings, engagement, relationships, meaning, and accomplishment) or the overall well-being score. The analysis indicates that the calculated *F*-values for all elements and the total score are lower than the critical *F*-values, which are 2.70 at the 0.05 significance level and 3.98 at the 0.01 significance level. These results suggest that academic level does not significantly affect the well-being dimensions or overall well-being as measured in this study.

**Table 6:** One-way ANOVA analysis of level of well-being based on academic level (*n* = 2,927).

Variables according to education	Higher than University (*n* = 185)		University (*n* = 1969)		Secondary (*n* = 658)		Intermediate (*n* = 72)		Primary (*n* = 23)		Uneducated (*n* = 20)		*f*	*p*-value	*Sig.*
	*M*	SD	*M*	SD	*M*	SD	*M*	SD	*M*	SD	*M*	SD			
Positive feelings	6.893	2.151	6.842	2.169	6.835	2.282	6.671	2.044	6.289	2.062	6.633	1.847	0.431	0.827	*NS*
Engagement	6.726	2.056	6.627	1.997	6.620	1.997	6.467	1.792	6.898	1.308	6.616	1.435	0.270	0.930	*NS*
Relationships	6.981	2.141	6.842	2.134	6.924	2.121	6.893	1.941	6.811	1.855	7.016	1.602	0.278	0.926	*NS*
Meaning	7.021	2.346	6.937	2.264	6.925	2.341	6.949	2.097	6.826	2.206	6.95	1.495	0.065	0.997	*NS*
Accomplishment	6.668	1.986	6.616	1.959	6.644	1.970	6.625	1.815	6.608	1.662	6.566	2.032	0.044	0.999	*NS*
Total score	6.857	2.136	6.772	2.104	6.789	2.142	6.721	1.937	6.686	1.818	6.756	1.682	0.332	0.894	*NS*

NS, not significant.

Table 7 indicates that there are no statistically significant differences attributable to marital status across the five elements of well-being in the PERMA model (positive feelings, engagement, relationships, meaning, and accomplishment) or the overall well-being score. The analysis shows that the calculated *F*-values for all elements and the total score are lower than the critical *F*-values, which are 2.70 at the 0.05 significance level and 3.98 at the 0.01 significance level. These findings suggest that marital status does not significantly influence the well-being dimensions or the overall well-being score as measured in this study.

**Table 7:** One-way ANOVA analysis of level of well-being based on marital status (*n* = 2,927).

Variables according to marital status	Single (*n* = 1,630)		Married (*n* = 1,186)		Divorced (*n* = 85)		Widowed (*n* = 26)		*f*	*p*-value	*Sig.*
	*M*	SD	*M*	SD	*M*	SD	*M*	SD			
Positive feelings	20.452	6.767	20.495	6.284	21.282	6.474	21.346	6.286	0.576	0.631	*NS*
Engagement	19.832	6.017	19.895	5.876	20.823	5.849	20.269	6.885	0.780	0.505	*NS*
Relationships	20.576	6.384	20.631	6.333	21.129	6.491	20.807	6.280	0.216	0.885	*NS*
Meaning	20.822	6.861	20.755	6.774	21.223	6.894	22.076	7.567	0.427	0.733	*NS*
Accomplishment	19.831	5.918	19.889	5.797	20.717	6.115	19.384	5.586	0.675	0.567	*NS*
Total score	150.88	31.634	151.12	30.927	154.87	32.278	152.73	31.13	0.459	0.711	*NS*

NS, not significant.

Table 8 presents the five elements of well-being in the PERMA model (positive feelings, engagement, relationships, meaning, and accomplishment) as well as the overall well-being score. The analysis reveals that the calculated *F*-values for most dimensions and the total score are lower than the critical *F*-values, which are 2.70 at the 0.05 significance level and 3.98 at the 0.01 significance level, indicating no statistically significant differences attributable to the family size variable. However, an exception was found for the “meaning” dimension, where the differences were statistically significant. To identify the direction of these differences based on family size, the LSD test for pairwise comparisons was conducted, as shown in Table 9.

**Table 8:** One-way ANOVA analysis of level of well-being based on family size (*n* = 2,927).

Variables according to family size	1–2 members (*n* = 291)		3–4 members (*n* = 677)		More than (5) members (*n* = 2059)		*f*	*p*-value	*Sig.*
	*M*	SD	*M*	SD	*M*	SD			
Positive feelings	20.498	6.639	20.670	6.296	20.455	6.626	0.243	0.784	*NS*
Engagement	20.027	5.895	20.036	6.038	19.830	5.952	0.354	0.702	*NS*
Relationships	20.436	6.153	21.140	6.082	20.495	6.464	2.447	0.087	*NS*
Meaning	20.728	6.862	21.514	6.581	20.635	6.886	3.765	0.023	0.05*
Accomplishment	19.900	5.859	20.143	5.855	19.798	5.878	0.780	0.458	*NS*
Total score	151.110	31.770	152.792	31.608	150.641	31.224	1.061	0.347	*NS*

NS, not significant, * statistically significant at *p* < 0.05 level.

**Table 9:** Analysis of post-hoc comparisons according to the variable of family size.

Variables	Family size	Mean differences					
		*M*	*P*	*M*	*P*	*M*	*P*
		1–2 members		3–4 members		More than (5) members	
Meaning	1–2 members			0.786	0.109	0.0927	0.828
	3–4 members					0.878*	0.006
	More than (5) members						

* Statistically significant at *p* < 0.05 level.

Table 9 indicates that there are statistically significant differences at the 0.05 significance level among the study sample based on the variable of family size in the “meaning” dimension. Specifically, significant differences were found between the categories “three to four individuals” and “more than five individuals,” with the latter group scoring higher. The group with “more than five individuals” had a mean score of 20.635 and a standard deviation of 6.886. However, no significant differences were observed between the categories “one to two individuals” and “three to four individuals,” nor between “one to two individuals” and “more than five individuals.”

Table 10 presents the five elements of well-being in the PERMA model (positive feelings, engagement, relationships, meaning, and accomplishment) and the total well-being score. The analysis shows that the calculated *F*-values for positive feelings, relationships, meaning, accomplishment, and the overall well-being score are lower than the critical *F*-value (2.70 at the 0.05 significance level and 3.98 at the 0.01 significance level), indicating no statistically significant differences attributable to the job variable in these elements. However, statistically significant differences were identified in “engagement” and the overall well-being score. To determine the direction of these differences based on the job variable, the LSD test for post-hoc comparisons was conducted, as presented in Table 11.

**Table 10:** One-way ANOVA analysis of level of well-being based on job (*n* = 2,927).

Variables according to job	Working (*n* = 1,107)		Not working (*n* = 562)		Student (*n* = 1,142)		Retired (*n* = 116)		*f*	*p*-value	*Sig.*
	*M*	SD	*M*	SD	*M*	SD	*M*	SD			
Positive feelings	20.696	6.660	20.464	6.633	20.302	6.466	20.793	6.210	0.759	0.517	*NS*
Engagement	20.286	6.043	19.934	6.105	19.516	5.785	19.586	6.041	3.241	0.020	*0.05*
Relationships	20.729	6.486	20.674	6.415	20.437	6.219	21.025	6.367	0.591	0.621	*NS*
Meaning	21.086	6.842	20.718	6.804	20.578	6.740	21.094	7.689	1.141	0.331	*NS*
Accomplishment	20.177	5.878	19.606	5.981	19.719	5.799	19.870	5.938	1.634	0.179	*NS*
Total score	153.322	32.710	150.69	31.527	149.11	29.828	151.74	30.990	3.441	0.016	*0.05*

NS, not significant, * statistically significant at *p* < 0.05 level.

**Table 11:** Analysis of post-hoc comparisons according to the variable of job.

Variables	Family size	Mean differences							
		*M*	*P*	*M*	*P*	*M*	*P*		
		Working		Not working		Student		Retired	
Engagement	Working			0.352	0.254	0.769*	0.002	0.700	0.228
	Not working					0.417	0.174	0.347	0.567
	Student							0.0695	0.905
	Retired								
Total	Working			2.628	0.105	4.211*	0.001	1.581	0.605
	Not working					1.582	0.327	1.047	0.743
	Student							2.630	0.389
	Retired								

* Statistically significant at *p* < 0.05 level.

Table 11 reveals statistically significant differences at the 0.050.050.05 significance level among the study sample based on the job variable in the “engagement” element. Specifically, significant differences were found between the categories “works” and “student,” favoring the “works” category, which had a mean score of 20.286 and a standard deviation of 6.043. No significant differences were observed between the categories “works” and “not working,” “works” and “retired,” “not working” and “student,” “not working” and “retired,” or “student” and “retired.”

Similarly, statistically significant differences were observed in the total well-being score based on the job variable between the “works” and “student” categories, again favoring the “works” category, which had a mean score of 153.322 and a standard deviation of 32.710. However, no significant differences were found between the categories “works” and “not working,” “works” and “retired,” “not working” and “student,” “not working” and “retired,” or “student” and “retired.”

The 0.05 significance level and 3.98 at the 0.01 significance level for “engagement,” “relationships,” “achievement,” and the total well-being score, indicating no statistically significant differences attributable to the income level variable for these elements. However, significant differences were identified in “positive feelings” and “meaning.” To determine the direction of these differences based on income level, the LSD test for post-hoc comparisons was conducted, as presented in Table 12.

**Table 12:** One-way ANOVA analysis of level of well-being based on income level (*n* = 2,927).

Variables according to income level	Less than 15 thousand Riyals (*n* = 1862)		Between 15 to 25 thousand Riyals (*n* = 612)		More than 25 thousand Riyals (*n* = 136)		Other (*n* = 317)		*f*	*p*-value	*Sig.*
	*M*	SD	*M*	SD	*M*	SD	*M*	SD			
Positive feelings	20.508	6.591	20.075	6.772	20.845	6.341	21.126	5.944	2.950	0.019	*NS*
Engagement	19.844	6.075	19.743	5.976	20.514	5.395	20.129	5.437	0.828	0.478	*0.05*
Relationships	20.686	6.290	20.220	6.753	20.308	6.606	21.028	5.892	1.412	0.237	*NS*
Meaning	20.826	6.869	20.318	7.024	21.176	6.412	21.545	6.339	2.418	0.064	*NS*
Accomplishment	19.876	5.877	19.575	6.181	20.588	5.456	20.094	5.357	1.349	0.257	*NS*
Total score	151.085	31.803	149.477	31.986	153.147	28.918	153.271	28.018	1.249	0.290	*0.05*

NS, not significant, * statistically significant at *p* < 0.05 level.

Table 13 indicates statistically significant differences at the 0.050.050.05 significance level among the study sample based on the income level variable in the “positive feelings” dimension. Specifically, significant differences were observed between the categories “15,000 to 25,000 riyals” and “others,” favoring the “others” category, which had a mean score of 21.126 and a standard deviation of 5.944. No significant differences were found among the remaining income level categories. Similarly, significant differences were identified in the “meaning” dimension based on income level, with differences between the “15,000 to 25,000 riyals” category and “others,” again favoring the “others” category, which had a mean score of 21.545 and a standard deviation of 6.339. No significant differences were found among the other categories.

**Table 13:** Analysis of post-hoc comparisons according to the variable of income level.

Variables	family size	Mean differences							
		*M*	*P*	*M*	*P*	*M*	*P*		
		Less than 15 thousand Riyals (*n* = 1862)		Between 15 to 25 thousand Riyals (*n* = 612)		More than 25 thousand Riyals (*n* = 136)		Other (*n* = 317)	
Positive Feelings	Working			0.432	0.157	0.337	0.562	0.618	0.121
	Not working					0.770	0.215	1.051*	0.021
	Student							0.280	0.671
	Retired								
Meaning	Working			0.508	0.111	0.349	0.564	0.719	0.083
	Not working					0.857	0.185	1.227*	0.009
	Student							0.369	0.598
	Retired								

* Statistically significant at *p* < 0.05 level.

## 4 Discussion

The current study aims to assess the level of well-being in the Saudi population according to various demographic variables. The results indicate that the overall level of well-being among the Saudi population is high. This can be attributed to the efforts of the Saudi government to enhance well-being, particularly through initiatives linked to Saudi Vision 2030. These social and economic developments have directly and indirectly contributed to improved well-being (Alenezi, 2022; Mohammad et al., 2024).

The findings may also reflect the Kingdom’s commitment to promoting mental health by enacting legislation aligned with World Health Organization (WHO) recommendations and the United Nations Principles for the Protection of Persons with Mental Illness. Notably, Saudi Arabia allocates 4% of its total health expenditure to mental health care, exceeding the global average of less than 0.2%.

This result reveals consistency with Hamdan-Mansour et al. (2015), which showed that approximately 50% of Saudi patients with chronic diseases reported high life satisfaction and moderate psychological distress. However, the findings diverge from those of Abu-Helalah et al. (2022), who reported lower quality-of-life and mental health scores among colorectal cancer patients in Saudi Arabia compared to regional and international figures. They also differ somewhat from Al Mutair et al. (2021) study, which found that emotional well-being in the KSA was moderate.

Gender differences in well-being levels were negligible, suggesting no discrimination in service provision between males and females in Saudi Arabia. Both genders exhibited similar rankings for PERMA model dimensions. This outcome reflects the principles of justice, consultation, and equality embedded in the Kingdom’s governance, as outlined by Van Eijk (2010). However, this result contrasts with Al Mutair et al. (2021), which identified factors like age, gender, and socioeconomic status as significant influences on emotional health during the COVID-19 pandemic.

Age differences were evident, with younger individuals (15–24 years) prioritizing “meaning” while older adults (65+ years) emphasized “relationships.” This likely reflects shifts in life priorities, where younger individuals seek purpose through work and achievement, while older individuals focus on forming meaningful relationships. These findings align with Hallaj (2020) who emphasized the importance of family relationships for the elderly, and with Al Mutair et al. (2021) study which highlighted age as a key determinant of emotional health.

Educational levels also influenced well-being dimensions. “Meaning” ranked highest among all educational categories except for the primary education group, where “engagement” took precedence. Individuals with no formal education prioritized “relationships.” This reflects the differing life perspectives associated with educational attainment. Uneducated individuals may value social connections as a source of self-worth, while educated individuals derive meaning from their accomplishments. Melero et al. (2023), similarly emphasize the multifaceted relationship between education and well-being. In this same fining, Yafi et al. (2021) also pointed out that educational accomplishment shows a close and complex relationship with various factors, including student motivation, academic satisfaction, and general emotional well-being within the educational environment, underscoring the multifaceted nature of the relationship between education and mental health.

Employment status was another key variable, with the highest well-being levels observed among working individuals, followed closely by retirees. Workers strive for personal growth and achievement, while retirees find fulfillment in life accomplishments and relationships. This is due to the nature of life and the circumstances that people experience in each of these two categories. On the one hand, workers often strive to achieve specific life goals and to overcome the obstacles they face to attain permanent progress, continuity, and personal growth. On the other hand, retirees have reached a stage of satisfaction with life and the work they have previously done, and the accomplishment of life goals. They can share their achievements and establish positive, fulfilling relationships with others, which are impregnated with security and mutual respect as a result of their previous life experiences. This raises the level of well-being for both groups. This is confirmed by Fugl-Meyer et al. (2002) study which found a positive correlation between life satisfaction and age.

The study’s results also demonstrate that the level of well-being in all categories of the variable “income level” was high, with convergence in the order of the dimensions of the PERMA model in each income level category. This is because the happiness an individual can feel is not related to his income level (Helliwell et al., 2017). His income may be low, but he can still experience high levels of satisfaction, joy, optimism, and self-realization. This is consistent with Ryff (2023) argument regarding the characteristics that contribute to the psychological health of the individual, the most important of which are self-acceptance, personal development, and a sense of the meaning and purpose of life. Ryff (2023) did not mention income as being among those characteristics. However, Owusu (2023) pointed out that there is a direct relationship between financial satisfaction and psychological well-being. Still, satisfaction is not related to income level. The income may be low, but an individual can still feel financially satisfied.

Regarding the family size variable, the results showed that the average overall well-being score for the different family size categories was high. The family size of between three and five individuals scored highest in well-being, with slight differences between the other categories. This result can be interpreted as signifying that family life generally gives a feeling of stability, a meaning to life, and the positive feelings associated with every accomplishment of one of its members. Family size was positively associated with well-being, with families of three to five members reporting the highest scores. Family life generally fosters stability, meaning, and shared accomplishments, contributing to higher well-being levels (Barendregt et al., 2015).

As for the nationality variable, the results showed no effect of this variable on the level of well-being, as the order of the dimensions of the PERMA model did not differ in any of the nationality categories. Moreover, the category of meaning came in the first place, and the category of accomplishment came in the last place among the two nationality categories in the current research. This is perhaps due to the nature of life in Saudi and the equality of privileges that individuals obtain on Saudi soil, regardless of their nationality, which positively affects their psychological well-being (Albassam, 2021).

The study remains significantly important and original since it is the first to examine well-being levels in the Saudi population through the PERMA model, with a focus on related demographic variables using the well-being. It has potential value for understanding the priorities and concepts of well-being among a range of demographic variables in the Saudi population.

However, there are potential limitations. First, though the sample size was nationally representative from a statistical perspective, the sample itself showed demographic characteristics that do not mirror the proportions in the entire country. This brings forth the qualitative limitation of the sample, in that it fails to represent the actual variability in the population with reference to other factors such as demographic characteristics. Indeed, this limits the external validity and generalizability of the present findings. Therefore, readers are recommended to exercise caution when interpreting these findings and applying them to other settings and samples. Other methodological limitations are that the study sample did not equally represent all Saudi regions and the limited number of similar regional studies restricted comparisons of findings. With regard to assessing the core variable, that is well-being, the study was limited by reliance on a single measurement scale and a lack of comparative tools or benchmarks. Furthermore, perceptions of well-being may vary by several personal and temporal factors such as time taken for completion, time of the day when the questionnaire is completed, and mental state at that moment. Unfortunately, we did not control for these variables when collecting our data. These limitations need to be considered when interpreting and generalizing the findings, and future studies need to be mindful of these factors.

## 5 Conclusion

This study concludes that the well-being of the sample population in Saudi Arabia is generally high, with no apparent gender-based disparities in service provision. Age differences influence well-being, with workers and retirees reporting the highest levels. Despite varying income levels, well-being remains consistently high across different income categories, indicating the success of the initiatives aligned with Saudi Vision 2030. Legislative measures promoting mental health, alongside equal privileges regardless of nationality, contribute positively to psychological well-being in the country. The findings suggest that Saudi Arabia is committed to enhancing the well-being of its citizens, especially in light of Vision 2030 and its associated social and economic developments. The study underscores the importance of ongoing efforts to ensure equitable access to services and promote mental health, reflecting a broader commitment to societal well-being in Saudi Arabia.

The study’s implications propose several avenues for further research and action. Firstly, it underscores the importance of continuing efforts in the KSA to enhance psychological well-being through diverse activities and supportive programs. Additionally, there is a recommendation for the Ministry of Information to design educational initiatives aimed at raising awareness about psychological well-being’s significance across various life domains. Furthermore, attention should be directed toward creating conducive job opportunities to bolster individuals’ psychological well-being. There is also a call for advanced studies to be conducted to devise customized programs positioning with technological advancements to foster psychological well-being throughout Saudi society. Specifically, investigations are needed to explore the correlation between psychological well-being and job satisfaction among workers across different ministries, as well as its relationship with individuals’ intellectual pursuits.

Looking forward, future research should broaden its scope by examining well-being in conjunction with other variables such as health factors, conducting longitudinal studies to understand well-being dynamics and their determinants, employing diverse metrics for measurement and comparison, and fostering community well-being development initiatives.

## Data Availability

The original contributions presented in the study are included in the article/supplementary material, further inquiries can be directed to the corresponding author.

## References

[ref1] Abu-Helalah M. Mustafa H. Alshraideh H. Alsuhail A. I. Almousily O. A. Al-Abdallah R.. (2022). Quality of life and psychological wellbeing of colorectal cancer survivors in the KSA. Asian Pac. J. Cancer Prev. 23, 1301–1308. doi: 10.31557/APJCP.2022.23.4.1301, PMID: 35485689 PMC9375610

[ref2] Ahrens C. J. C. Ryff C. D. (2006). Multiple roles and well-being: Sociodemographic and psychological moderators. Sex Roles 55, 801–815. doi: 10.1007/s11199-006-9134-8

[ref3] Al Mutair A. Alhajji M. Shamsan A. (2021). Emotional wellbeing in Saudi Arabia during the COVID-19 pandemic: a national survey. Risk Manag. Healthc. Policy 14, 1065–1072. doi: 10.2147/RMHP.S279716, PMID: 33737847 PMC7966358

[ref4] Albassam B. A. (2021). Achieving sustainable development by enhancing the quality of institutions in Saudi Arabia. Int. Sociol. 36, 439–463. doi: 10.1177/0268580921993327

[ref5] Alenezi D. (2022). Vision 2030: Leadership Styles, Readiness for Transformation and Faculty Satisfaction in Saudi Arabia. Australia: The University of Newcastle.

[ref6] Al-Hendawi M. Alodat A. Al-Zoubi S. Bulut S. (2024). A PERMA model approach to well-being: a psychometric properties study. BMC Psychol. 12:414. doi: 10.1186/s40359-024-01909-0, PMID: 39080800 PMC11290191

[ref7] AlzaidAlsharif H. I. (2022). The transition of Saudi Arabia from a resource-based economy to a knowledge-based economy: developing human, social, and moral capital in the 21st century. Freibadstr, München: LINCOM GmbH.

[ref001] Ayse E. B. (2018). Adaptation of the PERMA well-being scale into Turkish: Validity and reliability studies. Educ. Res. Rev. 13, 129–135.

[ref8] Barendregt C. Van der Laan A. Bongers I. Van Nieuwenhuizen C. (2015). Stability and change in subjective quality of life of adolescents in secure residential care. J. Forensic Psychiatry Psychol. 26, 493–509. doi: 10.1080/14789949.2015.1034751

[ref002] de Carvalho T. F. de Aquino S. D. Natividade J. C. (2023). Flourishing in the Brazilian context: Evidence of the validity of the PERMA-profiler scale: PERMA-profiler Brazil. Curr. Psychol. 42, 1828–1840.

[ref9] Dhanabhakyam M. Sarath M. (2023). Psychological wellbeing: Asystematic literature review. Int. J. Adv. Res. Sci. Commun. Technol. 3, 603–607. doi: 10.48175/IJARSCT-8345

[ref10] Diener E. Oishi S. Tay L. (2018). Advances in subjective well-being research. Nat. Hum. Behav. 2, 253–260. doi: 10.1038/s41562-018-0307-6, PMID: 30936533

[ref11] Dr K. Shukla K. (2023). Psychological well-being related to life satisfaction and happiness in among relationships. Quaderns Journal. vol. 11, 289–294.

[ref12] Fan L. Babiarz P. (2019). The determinants of subjective financial satisfaction and the moderating roles of gender and marital status. Fam. Consum. Sci. Res. J. 47, 237–259. doi: 10.1111/fcsr.12297

[ref13] Feng X. Lu X. Li Z. Zhang M. Li J. Zhang D. (2020). Investigating the physiological correlates of daily well-being: a PERMA model-based study. Open Psychol. J. 13, 169–180. doi: 10.2174/1874350102013010169

[ref14] Fugl-Meyer A. R. Melin R. Fugl-Meyer K. S. (2002). Life satisfaction in 18-to 64-year-old Swedes: in relation to gender, age, partner and immigrant status. J. Rehabil. Med. 34, 239–246. doi: 10.1080/165019702760279242, PMID: 12392240

[ref15] Gill A. Trask-Kerr K. Vella-Brodrick D. (2021). Systematic review of adolescent conceptions of success: Implications for wellbeing and positive education. Educ. Psychol. Rev. 33, 1553–1582. doi: 10.1007/s10648-021-09605-w

[ref003] Goodman F. R. Disabato D. J. Kashdan T. B. Kauffman S. B. (2018). Measuring well-being: A comparison of subjective well-being and PERMA. J. posit. psychol. 13, 321–332.

[ref16] Hallaj F. A. (2020). Quality Of Life of Community Dwelling Elders in Lattakia City, Syrian Arab Republic. Alex. Sci. Nurs. J. 22, 1–12. doi: 10.21608/asalexu.2020.206102

[ref17] Hamdan-Mansour A. M. Aboshaiqah A. E. Thultheen I. N. Salim W. M. (2015). Psychological wellbeing of Saudi patients diagnosed with chronic illnesses. Psychology. 6, 55–62. doi: 10.4236/psych.2015.61006

[ref004] Hassan E. M. (2016). Statistical Significance and Practical Significance in Research. Journal of Educational and Qualitative Studies and Research, Benha University, Faculty of Specific Education. 1, 20–32.

[ref18] Helliwell J. F. Huang H. Wang S. (2017). The social foundations of world happiness. World Happiness Rep. 8, 8–46.

[ref19] Hone L. C. Jarden A. Schofield G. Duncan S. (2014). Measuring flourishing: the impact of operational definitions on the prevalence of high levels of wellbeing. Int. J. Wellbeing. 4, 62–90. doi: 10.5502/ijw.v4i1.4

[ref20] Karasawa M. Curhan K. B. Markus H. R. Kitayama S. S. Love G. D. Radler B. T.. (2011). Cultural perspectives on aging and well-being: A comparison of Japan and the United States. Int. J. Aging Hum. Dev. 73, 73–98. doi: 10.2190/AG.73.1.d, PMID: 21922800 PMC3183740

[ref21] Kovich M. K. Simpson V. L. Foli K. J. Hass Z. Phillips R. G. (2023). Application of the PERMA model of well-being in undergraduate students. Int. J. Community Well-Being 6, 1–20. doi: 10.1007/s42413-022-00184-4, PMID: 36320595 PMC9607835

[ref22] Lindfors P. Berntsson L. Lundberg U. (2006). Factor structure of Ryff’s psychological well-being scales in Swedish female and male white-collar workers. Personal. Individ. Differ. 40, 1213–1222. doi: 10.1016/j.paid.2005.10.016

[ref23] Matud M. P. López-Curbelo M. Fortes D. (2019). Gender and psychological well-being. Int. J. Environ. Res. Public Health 16:3531. doi: 10.3390/ijerph16193531, PMID: 31547223 PMC6801582

[ref24] Melero S. Verdugo L. Sánchez-Sandoval Y. (2023). Psychological wellbeing in adult adoptees: current age and developmental tasks. Front. Psychol. 14:1190147. doi: 10.3389/fpsyg.2023.1190147, PMID: 37333601 PMC10273841

[ref25] Mohammad T. Kassem B. Mohammad S. (2024). “Employee Wellbeing and Quality of Life of Saudi Arabian Workers” in Employee wellbeing in the global south: a critical overview. Eds. Oruh, E. S. and Adisa, T. A. Cham: Springer Nature Switzerland. 121–144.

[ref006] Morgan K. (1970). Sample size determination using Krejcie and Morgan table. Kenya Projects Organization (KENPRO). 38, 607–610.

[ref26] Nurunnabi M. (2017). Transformation from an oil-based economy to a knowledge-based economy in Saudi Arabia: the direction of Saudi vision 2030. J. Knowl. Econ. 8, 536–564. Available at: https://link.springer.com/article/10.1007/S13132-017-0479-8

[ref27] Owusu G. M. Y. (2023). Predictors of financial satisfaction and its impact on psychological wellbeing of individuals. J. Humanit. Appl. Soc. Sci. 5, 59–76. doi: 10.1108/JHASS-05-2021-0101

[ref28] Park C. L. Kubzansky L. D. Chafouleas S. M. Davidson R. J. Keltner D. Parsafar P.. (2023). Emotional well-being: What it is and why it matters. Affect. Sci. 4, 10–20. doi: 10.1007/s42761-022-00163-0, PMID: 37070009 PMC10104995

[ref29] Ruggeri K. Garcia-Garzon E. Maguire Á. Matz S. Huppert F. A. (2020). Well-being is more than happiness and life satisfaction: a multidimensional analysis of 21 countries. Health Qual. Life Outcomes. 18, 1–16. doi: 10.1186/s12955-020-01423-y, PMID: 32560725 PMC7304199

[ref30] Rüppel F. Liersch S. Walter U. (2015). The influence of psychological well-being on academic success. J. Public Health. 23, 15–24. doi: 10.1007/s10389-015-0654-y

[ref31] Ryff C. D. (2023). “In Pursuit of Eudaimonia: Past advances and future directions” in Human flourishing: a multidisciplinary perspective on neuroscience, health, organizations and arts. eds. Heras M. L. Grau M. G. Rofcanin Y.. 9–31. doi: 10.1007/978-3-031-09786-7_2

[ref32] Seligman M. E. (2011). Flourish: a visionary new understanding of happiness and well-being. New York, NY: Simon and Schuster.

[ref33] Sweileh W. M. (2021). Contribution of researchers in the Arab region to peer-reviewed literature on mental health and well-being of university students. Int. J. Ment. Heal. Syst. 15:50. doi: 10.1186/s13033-021-00477-9, PMID: 34039394 PMC8153525

[ref34] Tansey T. N. Smedema S. Umucu E. Iwanaga K. Wu J.-R. Cardoso E. D. S.. (2018). Assessing college life adjustment of students with disabilities: Application of the PERMA framework. Rehabil. Couns. Bull. 61, 131–142. doi: 10.1177/0034355217702136

[ref35] Tay L. Li M. Myers D. Diener E. (2014). Religiosity and subjective well-being: An international perspective. In: Religion and Spirituality Across Cultures. Cross-Cultural Advancements in Positive Psychology. ed. Kim-Prieto, C. Dordrecht: Springer. 9, 163–175. doi: 10.1007/978-94-017-8950-9_9

[ref36] Thomson L. J. Lockyer B. Camic P. M. Chatterjee H. J. (2018). Effects of a museum-based social prescription intervention on quantitative measures of psychological wellbeing in older adults. Perspect. Public Health. 138, 28–38. doi: 10.1177/1757913917737563, PMID: 29130869

[ref37] Tov W. (2018). “Well-being concepts and components” in Handbook of subjective well-being. eds. Diener E. Oishi S. Tay L.. (UT: Noba Scholar), 1–15.

[ref38] Umucu E. Lee B. Iwanaga K. Kosyluk K. Blake J. Bezyak J.. (2021). Relationships between positive human traits and PERMA (positive emotion, engagement, relationships, meaning, and accomplishments) in student veterans with and without disabilities: a canonical correlation analysis. Rehabil. Res. Policy Educ. 35, 238–247. doi: 10.1891/RE-21-09

[ref39] Van Eijk E. (2010). “Sharia and national law in Saudi Arabia” in Sharia incorporated: a comparative overview of the legal systems of twelve Muslim countries in past and present, Ed. ott, J. M. Amsterdam: Amsterdam University Press. 139–180.

[ref40] Wagner L. Gander F. Proyer R. T. Ruch W. (2020). Character strengths and PERMA: Investigating the relationships of character strengths with a multidimensional framework of well-being. Appl. Res. Qual. Life. 15, 307–328. doi: 10.1007/s11482-018-9695-z

[ref41] World Health Organization (2022). Guide for integration of perinatal mental health in maternal and child health services: World Health Organization.

[ref007] Yamane T. (1967). Statistics, An Introductory Analysis, 2nd. New York, NY: Harper and Row.

[ref42] Yafi E. Tehseen S. Haider S. A. (2021). Impact of green training on environmental performance through mediating role of competencies and motivation. Sustain. For. 13:5624. doi: 10.3390/su13105624

